# Epidemiology and Prevention of Healthcare-Associated Infections in Geriatric Patients: A Narrative Review

**DOI:** 10.3390/ijerph18105333

**Published:** 2021-05-17

**Authors:** Maria Luisa Cristina, Anna Maria Spagnolo, Luana Giribone, Alice Demartini, Marina Sartini

**Affiliations:** Department of Health Sciences, University of Genoa, 16132 Genoa, Italy; cristinaml@unige.it (M.L.C.); luana.giribone@libero.it (L.G.); alice.demartini23@gmail.com (A.D.); sartini@unige.it (M.S.)

**Keywords:** healthcare-associated infections, elderly, epidemiology

## Abstract

Demographic studies show that life expectancy is increasing in developed countries; increased longevity has also increased the share of the older population with often concomitant chronic conditions. An ageing population and increased comorbidities lead to more complex pharmacological therapies (polypharmacy). The particular picture provided by chronic conditions and polypharmacy can lead to longer hospital stays and a greater need for healthcare. Elderly patients are identified as being in the high-risk group for the development of healthcare-associated infections (HAIs) due to the age-related decline of the immune system, known as immunosenescence. Comorbid conditions can often complicate infections, diminishing our ability to treat them effectively. Respiratory tract infections are the most common healthcare-associated infections, followed by urinary tract infections. HAIs in geriatric patients are responsible for longer hospital stays, extended antibiotic therapy, significant mortality, and higher healthcare costs. This is because the microorganisms involved are multidrug-resistant and, therefore, more difficult to eliminate. Moreover, geriatric patients are frequently transferred from one facility (nursing homes, skilled nursing facilities, home care, and other specialty clinics) to another or from one hospital ward to another; these transitions cause care fragmentation, which can undermine the effectiveness of treatment and allow pathogens to be transferred from one setting to another and from one person to another. Multifactorial efforts such as early recognition of infections, restricted use of invasive devices, and effective infection control measures (surveillance, isolation practices, hand hygiene, etc.) can contribute to significant reduction of HAIs in geriatric patients.

## 1. Introduction

Demographic studies show that life expectancy is increasing in developed countries due to improved social, economic, and health conditions and decreased fertility. In 2050, the population aged 65 and over is projected to be 83.7 million in the United States, almost twice its elderly population in 2012 [1]. Italy is one of the countries with the oldest populations. In 2002, the share of the older population was 18.7%. In 2020, it is 23.2%, and set to keep increasing [2].

As the prevalence study by Cairns et al. [3] has shown, the risk of contracting healthcare-associated infections (HAIs) increases linearly with increasing age. More specifically, HAI prevalence is 11.5% in patients aged over 85 years, while it is 7.4% in patients aged under 65 years.

The main microorganisms involved in HAIs in geriatric patients are the following: *E. coli*, *S. aureus*, *Klebsiella* spp., *P. aeruginosa*, *C. difficile*, *Acinetobacter* spp., *Enterococcus* spp., *Candida* spp., *Enterobacter* spp., and *Proteus* spp. [4,5,6,7,8]. Many of these microorganisms are multidrug-resistant organisms (MDRO) [9,10,11,12,13].

Multidrug resistance is one of the biggest problems in nursing homes. In 2013, the CDC (Centers for Disease Control and Prevention, Atlanta, GA, USA) identified the 18 top drug-resistant microorganisms, of which a staggering 11 were more prevalent in nursing home patients [14].

In 2017, the Istituto Superiore di Sanità (ISS) launched the PAMURSA project. The goal was to analyze the circulation of MDROs among older people residing in a select group of nursing homes. In particular, the observed prevalence of colonization (extended-spectrum β-lactamase-producing *Enterobacteriaceae* 57.3%, MRSA 17.2%, *C. difficile* 5.1%) was similar or higher than that detected in hospitals [6]. Identifying and isolating patients colonized with MDROs is relatively easy in intensive care units, but far more complicated in nursing homes, where patients regularly dine together and participate in communal activities [15,16,17].

This paper is a narrative review and summarizes/synthesizes what has been written on the topic, without collecting or analyzing any primary data [18,19].

## 2. HAIs in the Elderly

### 2.1. HAIs in Healthcare Facilities

The prevalence study by Cairns et al. [3] has shown how respiratory tract infections are the most common healthcare-associated infections, followed by urinary tract infections [4].

Although only one out of every 1000 cases necessitates hospitalization, this rate is 12 in 1000 for patients over 75 years and 33 in 1000 for nursing home patients [20].

Pneumonia is one of the most severe respiratory tract infections. Among hospital-acquired pneumonias (HAP), ventilator-associated pneumonia (VAP) is the most clinically relevant in geriatric patients. Chronic obstructive pulmonary disease predisposes bacterial pneumonia caused by *Haemophilus influenzae*, *Moraxella catarrhalis*, and *Legionella pneumophila* [20].

Viruses including influenza, rhinovirus, and human metapneumovirus can be detected frequently [21,22,23]. An increased rate of co-infections in the elderly with influenza admitted to the ICU has been reported and is associated with 28-day mortality and hospital mortality [20,24].

The pathogens involved are mainly gram-negative bacteria (many of which are multidrug-resistant), including *P. aeruginosa*, *Klebsiella* spp., and *Citrobacter* spp. These bacteria can invade the lower respiratory tract by aspiration of oropharyngeal organisms. Some clinical conditions, such as severe underlying disorders, increase bacterial adhesion to the oropharyngeal mucosa. People with impaired swallowing or dysphagia who have been intubated or have undergone surgery are at high risk of aspiration and are thus more likely to contract aspiration pneumonia. Undergoing invasive healthcare procedures is the main HAP risk factor. Mechanical ventilation and the bacterial colonization of healthcare personnel’s hands also increase a patient’s risk of infection [25].

Urinary tract infections (UTIs) are the second most common healthcare-associated infection. UTIs’ aetiology varies between non-catheterized patients (or those catheterized for less than 30 days) and patients with a chronic, indwelling catheter (those catheterized for more than 30 days). This is because catheters have been demonstrated to be a common source of infection. In the former case, UTIs are usually monomicrobial, and *Escherichia coli* is the most common aetiologic agent. In the latter case, the infection is usually polymicrobial, with a variable bacterial composition [26,27]. However, urinary catheter colonization frequently occurs after hospitalization [20].

A study published in 2015 [28] shows that the prevalence of catheterized nursing home residents in the United States ranges from 5% to 22%. A comparison of the infection rates between catheterized and non-catheterized residents shows 6.2 clinically defined UTIs per 1000 days in catheterized patients versus 2.8 UTIs/1000 days in non-catheterized patients.

Multidrug resistance, particularly in gram-negative bacteria, is challenging, and may cause treatment failures of urinary tract infections. These bacteria include extended-spectrum beta-lactamase (ESBL) production and/or carbapenem-resistant *Enterobacteriaceae*, and recently emerging colistin-resistant gram-negative bacilli [20,29].

In addition to catheterization, predisposing factors for urinary tract infection in older patients include neurogenic bladder (Alzheimer’s and Parkinson’s), diabetes mellitus (especially in the case of poor glycaemic control), lack of oestrogen, post-void residual urine, and incontinence in postmenopausal women. Other urinary tract conditions include chronic prostatitis, prostate hypertrophy, cystocele, and obstructions (kidney stones or neoplasms) [28].

Environmental predisposing factors include contaminated patient areas and/or healthcare devices, healthcare workers’ poor hand hygiene, and poor hygiene in dependent patients [26].

A study by Sugishita [30] showed that the majority of 76 patients (mean age: 84.8 years) presenting with UTIs had a condition that predisposed them to infection. In particular, 39.5% had cerebrovascular disorders, 3.9% had neurodegenerative disorders, and 17.1% had diabetes mellitus. Infection was catheter-associated in 13.2% of cases. In any case, hospitalization is considered a risk factor for bacteriuria [28].

Skin and soft tissue infections are other common HAIs that can occur in older patients following the development of pressure ulcers. Primary bacterial infections are often caused by microorganisms that are part of the microbiota of human skin and mucous membranes, such as *Staphylococcus aureus* and group A β-hemolytic streptococci. Existing wounds can be secondarily infected by microorganisms transferred from other patients through the hands of healthcare personnel or the environment [31,32].

Pressure ulcers are frequent but potentially preventable in older people with compromised functional independence requiring complete bed confinement. In the absence of appropriate preventive measures, pressure ulcers during hospitalization can become complications that lead to extended inpatient stays for older patients and increased morbidity and mortality. Pressure ulcers are also indicators of the quality of care, as they can result from negligence by healthcare personnel [31,32].

Intrinsic and extrinsic factors cause pressure ulcers. Intrinsic factors include immobilization, extended bed confinement, malnutrition, bone fractures, musculoskeletal disorders, diabetes, use of restraints, obesity, compromised blood circulation, and skin failure (skin loses firmness and starts breaking down). Extrinsic factors include perspiration, shear force (patients sliding down the bed), contact with urine and exudate, friction, and pressure on skin over a bony prominence [32].

### 2.2. HAIs by Clostridium Difficile

Infections in older adults may not present in a typical manner [33]. An atypical presentation can lead to diagnostic delays or misdiagnosis in a population that is already at risk of higher morbidity and mortality and can ill afford delays in initiating antimicrobial therapy. Conversely, clinicians may overprescribe antibiotics, fearing that any given change in clinical status may indicate an infection, placing people at risk of *Clostridium difficile* infection (CDI), adverse side effects of the drug, or development of antimicrobial resistance [34].

The incidence of *C. difficile* infection is 4% to 20% higher in patients in healthcare facilities than in healthy adults. Over 80% of deaths due to CDI occur in those over 65 years of age [20,35]. The risk of *Clostridium difficile* infection can increase following the treatment of urinary tract infections with broad-spectrum antibiotics. 

Age, immunosuppression, severe comorbidity, nasogastric tube feeding, prolonged hospitalization, prolonged and combined antibiotic therapy, and proton pump inhibitor therapy are factors that contribute to *C. difficile* pathogenicity [15,36]. Recurrences are frequent and may occur in 20–30% of cases [20]. A recent analysis indicated that overall health status including infections, health care utilization, acute conditions in the past year, and frailty indicators are the most important determinants of CDI risk in an elderly population. Thus, an elderly person in critical condition will be more likely to have a severe CDI than a person with similar age or older without any comorbidity or previous hospitalization [20,37].

Table 1 shows the prevalence of HAIs according to site/wards of infection and, when reported, the most frequently responsible microorganisms. 

### 2.3. HAIs by Molds

Fungal infections, especially those caused by *Candida* spp., are widespread among hospitalized patients. The mortality rate for candidemia, for example, ranges between 36% and 63%, depending on the patient’s characteristics. The older population is especially vulnerable, probably because of various factors, including advanced age, high comorbidity, age-related physiological changes, polypharmacotherapy, and high colonization rates. However, data regarding epidemiology and candidemia outcomes in geriatric patients are inconsistent [43,44].

The retrospective study by Barchiesi et al. [43] shows how 30-day mortality was significantly higher in older patients (45%) than in younger ones (28%). The literature tells us that mortality in older patients is probably related to the lack of antifungal therapy or incorrect or delayed treatment. However, data remain controversial as there is no proof of a connection between antifungal medicines and higher survival rates [43,45,46].

### 2.4. COVID-19

In addition to these infections, both hospitals and nursing homes now have to deal with COVID-19, a major complication in the elderly, especially when it necessitates mechanical ventilation, which can cause VAP.

Comorbidity has been associated with higher mortality rates [47,48]. Age-related immunosenescence may explain older patients’ higher vulnerability to the infection and mortality rates [48,49]. A study carried out in Northern Italy in March 2020 [50] showed that 68% of patients had at least one comorbidity. Hypertension was the most frequent (49% of patients), followed by cardiovascular disorders (21%) and hypercholesterolemia (18%).

Common COVID-19 complications include bacterial infection, liver enzyme abnormalities, and acute respiratory distress syndrome [51]. In their study based in Wuhan, China, Zhou et al. [52] reported that half of non-survivors had a secondary infection. In particular, 32% of patients requiring invasive mechanical ventilation had ventilator-associated pneumonia (VAP).

The infection rate (Rt) was very high in nursing homes due to factors such as crowding, shared bathrooms, and common areas. Furthermore, healthcare workers were not suitably trained to control the infection. To this end, the CDC has issued guidelines for preventing and controlling COVID-19 [53]. Staffing shortages, frequent staff turnover, high resident-to-staff ratios, personal protective equipment (PPE) shortages, and inadequate infection prevention and control measures were the other main risk factors [54].

## 3. Physiopathological and Predisposing Factors in Older Patients

Increased longevity has also increased the proportion of the older population with often concomitant chronic conditions [55,56]. Multimorbidity (presence of two or more chronic conditions) is a global problem, and epidemiological data show that it affects between 55% and 98% of the elderly in disadvantaged social classes [56].

In Italy, more than half of the older population suffers from severe chronic conditions, and this rate will keep increasing [55,57]. In particular, 57% of the older population has arthritis, 55% has hypertension, 38% has respiratory problems, 17% has diabetes, 17% has cancer, and 16% has osteoporosis [55,56]. In Italy, the incidence of chronic conditions increases with age. In people aged between 55 and 65 years, the comorbidity rate ranges from 15% to about 25%. In people aged between 75 and 85 years, it exceeds 40% [56,58].

An ageing population and increased comorbidities lead to more complex pharmacological therapies (polypharmacy). A study on older patients admitted to six hospitals in Western Europe showed that they were taking an average of six medications. Moreover, this study highlighted how polypharmacy issues are more likely to emerge as more medications are prescribed [55,59].

The particular picture provided by chronic conditions and polypharmacy (and adverse reactions, if any) can lead to longer hospital stays and greater need for healthcare [41,60]. Moreover, conflicting specialist advice and the lack of coordination in the clinical and therapeutic management of the patient [55] can expose older patients to the risk of contracting healthcare-associated infections both in hospitals and nursing homes.

Elderly patients are identified as being in the high-risk group for the development of HAIs; indeed, infection is the primary cause of death in one third of individuals aged 65 years and over [34,38,42].

A secondary analysis of data from an international, observational, point prevalence study (EPIC II) revealed that out of infected patients who comprised 51.4% of the whole cohort, almost half (48.7%) were aged 65 and older [61]. There was no difference in disease severity among age groups (18–44, 45–64, 65–74, 75–84, 85), however, 85 years of age was an independent risk factor for admission to the ICU and hospital mortality. It has been shown that patients who are older than 85 had fewer bloodstream and central nervous system infections, but more intraabdominal infections as compared to younger patients. More gram-negative bacteria were isolated in those over 85 years of age, and in-hospital mortality was much higher in this age group compared to individuals younger than 65 [20,61].

An age-related factor to consider is the decline in immune function known as immunosenescence. Immunosenescence is present in all older adults to varying degrees, and there is a link between the degree of frailty and immunocompetency [62]. As the immune system ages and the normal capabilities of defense against infections and malignant or autoreactive cells declines, increased susceptibility to infections, malignancy, autoimmune disorders, and impaired wound repair follow [63,64]. Many older adults have mild degrees of immunosuppression as a result of immunosenescence, together with age related organ changes, comorbidities, geriatric syndromes, frailty, malnutrition, functional dysfunction and, polypharmacy, all of which affect the prognosis of geriatric patients with infectious diseases [20,62,65].

Another factor to take into account is that geriatric patients are frequently transferred from one facility (nursing homes, skilled nursing facilities, home care, and other specialty clinics) to another or from one hospital ward to another. These transitions cause care fragmentation, which can undermine the effectiveness of treatment and allow pathogens to be transferred from one setting to another and from one person to another. In other words, infection control in older patients varies depending on the healthcare service provided (e.g., assisted ventilation or catheter insertion), intrinsic factors, and the frequency of patient contact with healthcare workers and/or caregivers. Prompt and effective communication while transferring patients helps reduce the risk of healthcare-associated infections [66].

## 4. HAIs and Terminal Illness

Older patients with terminal illnesses may encounter complications, such as fever or infections associated with increased morbidity and mortality. In such cases, palliative care is preferred over more aggressive treatments, which may cause more suffering despite focusing on life-prolongation [67]. Infections occurring during end-of-life care may lead to overuse or misuse of antibiotics. Antimicrobials can cause harmful drug-drug interactions. Moreover, there is a higher risk of drug-resistant microorganisms and secondary infections (e.g., CDI) [67,68,69].

## 5. The Issue of Rehospitalization

About 5% of hospitalized geriatric patients are estimated to be readmitted to hospital within 30 days of discharge. This translates into a higher risk of contracting an infection for the patient and higher healthcare costs. Moreover, there is an increased risk of the patient bringing a community-acquired infection developed prior to readmission into a healthcare facility.

The most common infections associated with rehospitalization are surgical site infections (SSIs), *C. difficile* infections, UTIs, and central line-associated bloodstream infections (CLABSIs) [70]. Pre- and post-discharge measures must be taken to prevent or at least reduce the prevalence of these infections.

Before discharging a patient, it is crucial to inform them and their families and/or caregivers about hygiene practices that can help prevent infections and the pharmacological therapy (if any) to follow. It is also essential to ensure proper follow-up through prompt and direct communication between patient/family and treating physician/healthcare facility [71,72,73,74]. Factors contributing to communication breakdowns include insufficient information, ineffective communication methods, lack of time, distractions, lack of standardized procedures, and insufficient staffing [75].

A study by Hoffman et al. [70] has shown how important it is to correctly manage patients who have contracted a healthcare-associated infection after discharging them home. According to this study, readmission within 30 days of discharge for the same HAI treated during the first hospitalization was nearly 38% more frequent among patients discharged to their homes than those discharged to skilled nursing facilities (SNFs).

Many patients understandably prefer home care; however, some are not self-sufficient or cannot rely on adequate financial resources and support to achieve optimal recovery and proper healthcare at home. SNFs, on the other hand, can ensure adequate pharmacological treatment, rehabilitation, and monitoring of vital signs, fluid balance, and nutrition. This way, they can promptly identify and deal with any altered condition. According to this study, unless appropriate home healthcare can be ensured, it is advisable for older patients with comorbidities who have contracted a healthcare-associated infection to be discharged to a skilled nursing facility [66,70].

Various studies have assessed the risk factors for infection in patients receiving home healthcare (infections not included in HAI reports), and have identified indwelling catheters as the main cause [76,77,78].

Socioeconomic status is another risk factor that affects home hygiene and the possibility of accessing the necessary healthcare services. Patients with low socioeconomic status are at higher risk for contracting an infection during the post-discharge period [79]. In 2008, the Association for Professionals in Infection Control and Epidemiology, Inc. and the Centers for Disease Control and Prevention’s Healthcare Infection Control Practices Advisory Committee published the definitions for surveillance of infections in home healthcare, stressing how caregivers and the home environment can influence the patient’s risk of infection [76,80]. Ineffective communication among various clinicians involved in patient care may compromise patient safety and long-term recovery significantly [76].

## 6. HAIs Prevention

Prevention and control of HAIs are essential to reduce the impact of these infections and, more generally, to reduce the spread of MDROs. The main preventive measures to limit the occurrence of the most frequent HAIs described above are documented in the literature.

In the case of VAP, the main preventive strategy is to reduce mechanical ventilation, opting instead for non-invasive ventilation methods. Should it not be possible to avoid mechanical ventilation, it is essential to take measures to reduce the risk of VAP, such as daily interruption of sedation and spontaneous breathing trials. Moreover, a semi-recumbent position has been shown to be more effective in reducing aspiration risks in mechanically ventilated patients than a supine position. Moreover, a recent study has shown how oropharyngeal aspiration can help reduce VAP incidence by 45% [81,82].

The main preventive measure to reduce UTI risk is to avoid catheterization when not absolutely necessary, such as when collecting urine samples or in surgery not involving the bladder, female genitals, or gastrointestinal tract. Alternatives to catheterization, such as pharmacological or rehabilitative interventions, should be sought whenever possible to manage incontinence. Should it not be possible to avoid using a urethral catheter, it is essential to use the aseptic technique and minimize catheterization duration [83,84]. Patient education on using a hygienic catheterization technique and proper cleaning of reusable catheters is extremely important in preventing HHC UTIs.

Concerning SSTIs, it is essential to implement preventive measures that reduce the patient’s discomfort and healthcare costs. Such measures include changing the patient’s position in the event of extended bed confinement, minimizing prolonged pressure, keeping the skin clean and dry, and ensuring appropriate nutrition, personal hygiene, early rehabilitation, and caregiver education [32].

Regarding *C. difficile*, mechanisms to successfully prevent CDI include reducing modifiable risk factors and minimizing horizontal transmission of *C. difficile* spores between patients and the healthcare environment. The use of an Infection Control Committee approved disinfectant to reduce or eliminate *C. difficile* spores is an effective ‘facility-level strategy’.

Because CDI prevention is characterized by a bundled approach, it is difficult to quantify the individual impact of any one element; however, a number of patient-and facility-level strategies can be considered for CDI prevention. Robust hygiene strategies (in particular a strong hand hygiene program), diagnostic and antimicrobial stewardship, and particular prophylaxis maneuvers such as continuation of oral vancomycin or fidaxomicin alongside systemic antibiotics have all demonstrated benefit. It is of fundamental importance to isolate infected patients as soon as possible after the onset of their first symptoms [85,86,87]. Many randomized controlled trial studies assessed probiotics as an adjunct to standard antibiotic treatment for the prevention of *C. difficile* infection. The US Food and Drug Administration recently approved bezlotoxumab, the first human monoclonal antibody treatment for CDI [88].

In the spread of infections, a vehicle that must not be overlooked is that of the hands of healthcare personnel, which may become contaminated through contact with colonized/infected patients or with contaminated surfaces, soaps, creams, or water. Consequently, healthcare personnel have a decisive role to play in controlling this type of transmission. The risk of cross-contamination via hands has been demonstrated by the increasingly sophisticated techniques of molecular biology.

The application of control measures to the behavioral and procedural features of healthcare staff, such as strict adherence to hand-hygiene protocols, is extremely important in interrupting the patient-to-patient spread of various multidrug-resistant microorganisms [89].

## 7. Conclusions

Healthcare-associated infections in geriatric patients are responsible for longer hospital stays, extended antibiotic therapy, and higher healthcare costs. This is because the microorganisms involved are multidrug-resistant, and are therefore more difficult to eliminate. That is why infection prevention and control measures are crucial.

HAI rates can be reduced by ensuring appropriate hand hygiene and wearing PPE to protect patients from transmission of microorganisms from other patients. Other interventions that help protect patients from HAIs include suitable isolation strategies, sterilization and disinfection, appropriate antibiotic prophylaxis, minimizing invasive procedures to reduce the risk of endogenous infections, infection surveillance to identify and control outbreaks, improvement of healthcare practices, and continuing education for healthcare workers.

Moreover, environmental cleaning and disinfection are important components of a comprehensive strategy to control healthcare-associated infections [90].

From a broader perspective, the strategy of various healthcare systems must include tailored diagnostic, therapeutic, and rehabilitation pathways with a good cost/effectiveness ratio. Healthcare strategies should also increasingly empower patients and their families and promote care continuity between hospitals and the community [55,56].

## Figures and Tables

**Table 1:** Prevalence of healthcare-associated infections (HAIs) according to site/wards of infections and most frequent microorganisms.

Type of HAI	Prevalence	Reference	Healthcare Structure/Hospital Ward	Principal Organisms
Respiratory tract infections	35.8%	Serrano, 2015 [38]	LTCF	-
	31.0%	CCM, 2013 [39]	NH	-
	20.3%	CCM, 2016–2017 [8]	ICU	*P. aeruginosa*, *K. pneumoniae*, *S. aureus*, *E. coli*, *A. baumannii*
	20.0%	Cairns, 2011 [3]	Acute care hospital	-
	4.4%	Dwyer, 2013 [40]	Hospice	-
	2.6%	Sticchi, 2018 [41]	Geriatric ward	-
	2.4%	Dwyer, 2013 [40]	HHC	-
	2.2%		NH	
	2.2%	Koch, 2015 [42]	Tertiary hospitals	-
Urinary tract infections	35.8%	Serrano, 2015 [38]	LTCF	-
	31.0%	CCM, 2013 [39]	NH	-
	18.0%	CCM, 2016–2017 [8]	ICU	*E. coli*, *K. pneumoniae*, *E. faecalis*, *P. aeruginosa*, *P. mirabilis*
	17.9%	Cairns, 2011 [3]	Acute care hospital	-
	6.6%	Sticchi, 2018 [41]	Geriatric ward	-
	5.2%	Dwyer, 2013 [40]	NH	-
	3.6%		HHC	
	3.0%		Hospice	
	2.1%	Koch, 2015 [42]	Tertiary hospitals	-
Gastrointestinal infections	15.5%	Cairns, 2011 [3]	Acute care hospital	*C. difficile*
	8.49%	CCM, 2016–2017 [8]	ICU	*C. difficile*
	5.0%	CCM, 2013 [39]	NH	-
	1.3%	Sticchi, 2018 [41]	Geriatric ward	-
Soft and skin tissue infections	23.0%	CCM, 2013 [39]	NH	-
	17.0%	Serrano, 2015 [38]	LTCF	-
	10.9%	Cairns, 2011 [3]	Acute care hospital	-
	3.13%	CCM, 2016–2017 [8]	ICU	-
	2.0%	Dwyer, 2013 [40]	HHC	-
	1.6%		NH	
	0.5%		Hospice	
Bloodstream infections	18.3%	CCM, 2016–2017 [8]	ICU	*S. epidermidis*, *K. pneumoniae*, *S. aureus*, *E. coli*
	6.6%	Sticchi, 2018 [41]	Geriatric ward	-
	4.5%	Cairns, 2011 [3]	Acute care hospital	-
	0.5%	Koch, 2015 [42]	Tertiary hospitals	-
Surgical site infections	14.4%	CCM, 2016–2017 [8]	ICU	*S. aureus*, *K. pneumoniae*, *E. coli*, *P. aeruginosa*
	1.6%	Koch, 2015 [42]	Tertiary hospitals	-
	1.3%	Sticchi, 2018 [41]	Geriatric ward	-

LTCF, long-term care facilities; NH, nursing homes; ICU, intensive care units; HHC, home healthcare.
